# GammaTile: Comprehensive Review of a Novel Radioactive Intraoperative Seed-Loading Device for the Treatment of Brain Tumors

**DOI:** 10.7759/cureus.29970

**Published:** 2022-10-06

**Authors:** Chukwuyem Ekhator, Ijeoma Nwankwo, Elya Rak, Ariel Homayoonfar, Ekokobe Fonkem, Ramin Rak

**Affiliations:** 1 Neuro-Oncology, New York Institute of Technology College of Osteopathic Medicine, Old Westbury, USA; 2 Research, California Institute of Behavioral Neurosciences & Psychology, Fairfield, USA; 3 Neurosurgery, Friends Academy, Glen Cove, USA; 4 Neurosurgery, City University of New York (CUNY) Brooklyn College, Brooklyn, USA; 5 Neuro-Oncology, Barrow Neurological Institute, Phoenix, USA; 6 Neurosurgery, Neurological Surgery, P.C., Rockville Centre, USA

**Keywords:** start, radiation therapy, glioblastoma, brachytherapy, gammatile

## Abstract

GammaTile is a Food and Drug Administration (FDA)-licensed device consisting of four cesium-131 (Cs-131) radiation-emitting seeds in the collagen tile about the postage stamp size. The tiles are utilized to line the brain cavity immediately after tumor resection. GammaTile therapy is a surgically targeted radiation therapy (STaRT) that helps provide instant, dose-intense treatment after the completion of resection. The objective of this study is to explore the safety and efficacy of GammaTile surgically targeted radiation therapy for brain tumors. This study also reviews the differences between GammaTile surgically targeted radiation therapy (STaRT) and other traditional treatment options for brain tumors. The electronic database searches utilized in this study include PubMed, Google Scholar, and ScienceDirect. A total of 4,150 articles were identified based on the search strategy. Out of these articles, 900 articles were retrieved. A total of 650 articles were excluded for various reasons, thus retrieving 250 citations. We applied the exclusion and inclusion criteria to these retrieved articles by screening their full text and excluding 180 articles. Therefore, 70 citations were retrieved and included in this comprehensive literature review, as outlined in the Preferred Reporting Items for Systematic Reviews and Meta-Analyses (PRISMA) diagram. Based on the findings of this study, GammaTile surgically targeted radiation therapy (STaRT) is safe and effective for treating brain tumors. Similarly, the findings have also shown that the efficacy of GammaTile therapy can be enhanced by combining it with other standard-of-care treatment options/external beam radiation therapy (EBRT). Also, the results show that patients diagnosed with recurrent glioblastoma (GBM) exhibit poor median overall survival because of the possibility of the tumor returning. Therefore, combining STaRT with other standard-of-care treatment options/EBRT can improve the patient's overall survival (OS). GammaTile therapy enhances access to care, guarantees 100% compliance, and eliminates patients' need to travel regularly to hospitals for radiation treatments. Its implementation requires collaboration from various specialties, such as radiation oncology, medical physics, and neurosurgery.

## Introduction and background

Approximately 20%-40% of patients who develop primary cancer will develop brain metastases. Moreover, studies have estimated the diagnosis of about 200,000 new cases of brain metastases annually [1]. It is important to note that the metastatic lesion's size often determines the treatment approach. In this respect, symptomatic lesions larger than 2 cm in the location that can be accessed are typically resected, and stereotactic radiotherapy (SRT) is used to treat the surgical bed [1]. SRT is instrumental in improving local control over resection with a local recurrence-free survival rate of about 61%-72% at one year and is increasingly becoming the preferred standard of care [2]. However, radiation necrosis rates are vital for patients receiving SRT treatment in the postoperative setting, with the occurrence rate rising by 18.2% [2]. There is an unfulfilled need for the adjunctive radiation therapy approach to enhance the surgical bed control rate without brain injury's inherent risk to normal tissue. Moreover, there was a need for a therapy that could minimize the hair loss incidence as an unpleasant effect that could not negatively affect wound healing [3]. 

GammaTile is an FDA-licensed device consisting of four cesium-131 (Cs-131) radiation-emitting seeds in the collagen tile about the postage stamp size. The tiles line the brain cavity immediately after tumor resection [3]. Therefore, GammaTile therapy is a surgically targeted radiation therapy (STaRT) that helps provide instant, dose-intense treatment after the completion of resection [3,4]. Also, GammaTile is a biocompatible, permanent collagen tile implant that delivers radiation therapy to the location where a brain tumor has been removed [5]. Every tile is 2 cm × 2 cm and 4 mm thick containing four cesium-131 (Cs-131) titanium-encased sources [5]. After safely removing the tumor, the neurosurgeon places GammaTiles into the operative bed to cover the tumor cavity with tiles. The tile numbers utilized depend on the location and size of the tumor [6]. The procedure takes around five minutes for the surgeon to place the tiles at the end of the tumor removal surgery before closing the incision. After placing GammaTiles into the operative bed, they instantly deliver the uniform radiation dose to the target area. In this respect, 50% of the therapeutic dose is delivered in the first 10 days after surgery to help deter residual tumor cells from multiplying [7]. Also, 88% of the therapeutic dose is delivered within 30 days, with over 95% of the dose delivered within six weeks [3].

The structural counterbalance of the brain tissue sources is instrumental in preventing healthy tissues from the radiation's side effects [8]. Furthermore, the radiation's therapeutic dose is delivered over time, and the body naturally absorbs the tile. For patients suffering from recurrent brain metastases and meningiomas, researchers have established a significant decline in the treatment site reappearance when using GammaTile therapy compared to their earlier treatments [9]. Moreover, for patients suffering from recurrent glioblastomas (GBMs), GammaTile therapy has shown the possibility for enhanced general survival when comparing the surgery plus GammaTile therapy's effectiveness to other treatment modalities in various clinical studies [10]. GammaTile therapy is eligible to treat patients with newly diagnosed malignant and recurrent brain tumors. Most patients tend to experience fewer side effects compared to patients who have been treated using other radiation treatments [10]. The most common postoperative side effects experienced by some patients include vomiting, nausea, sleepiness, seizures, headache, and skin irritation [11].

The fundamental benefits of GammaTile surgically targeted radiation therapy include instant commencement of radiation treatment after tumor resection when the disease burden is minimal as opposed to waiting for the surgical wound to heal, which typically takes 3-4 weeks [12]. Moreover, this treatment approach optimizes radiation dosimetry, allowing the maximum dose in the tumor region and the lesser dose in the normal adjacent tissue [13]. Furthermore, it is used immediately after resection when there is visible surgical bed tissue to enable effective tile placement, enabling precise targeting of radiation therapy [2]. Moreover, patients hardly require returning to hospitals as outpatients for radiation treatment. GammaTile therapy is an effective and safe radiation option that hardly requires capital investment and helps eliminate the need for repeat treatments with other related caregivers and transportation burdens [14].

Similarly, this treatment option helps improve access to care and ensure 100% compliance, as patients can continue with their daily activities as they get their built-in radiation treatment [15]. Many clinical studies coupled with post-approval utilization have demonstrated the efficacy and safety of GammaTile surgically targeted radiation therapy [16]. This study aims to conduct a comprehensive literature review on the use of GammaTile surgically targeted radiation therapy (STaRT) for brain tumors.

## Review

Methods

This study explores GammaTile surgically targeted radiation therapy (STaRT) for brain tumors by reviewing various literature on this topic. Consequently, the systematic review studies the efficacy and effectiveness of GammaTile surgically targeted radiation therapy (STaRT) for brain tumors relative to other radiation therapies. In this respect, the study will be based on a comprehensive literature review. This section will cover the search strategy employed, inclusion and exclusion criteria, outline outcome measures, quality evaluation of eligible articles, and data extraction and analysis method used.

Search Strategy

The comprehensive literature review for this study reviewed journal materials on GammaTile surgically targeted radiation therapy (STaRT) for brain tumors in different database sources. To search for studies published from 2011 to date, the systematic review used the following electronic database searches: PubMed, EMBASE, ScienceDirect, Future Medicine, ELSEVIER, Google Scholar, and the Cochrane Controlled Trials Register for randomized controlled trials (RCT). Moreover, the literature review also analyzed cohort studies and randomized controlled trials, controlled trials, open-label studies, and uncontrolled trials.

Moreover, the researcher employed the search strategy that sought to identify journal materials published in the English language with the keywords "GammaTile therapy," "surgically targeted radiation therapy (STaRT)," "Radiation treatment after tumor resection," and "Brain tumor treatment." The search outcomes were unlimited and covered journal materials irrelevant to the study topic based on our search process. Consequently, it was essential to combine the keywords to initiate index terms, such as the efficacy of GammaTile surgically targeted radiation therapy (STaRT) for brain tumors and the effectiveness of GammaTile surgically targeted radiation therapy (STaRT) in treating brain tumors. This search strategy was instrumental in narrowing the search and enhancing the quality of the search outcomes that were relevant to the research topic. Moreover, the researcher also filtered the search outcomes using the keywords "GammaTile therapy," "surgically targeted radiation therapy (STaRT)," "Radiation treatment after tumor resection," and "Brain tumor treatment" in the journal articles' abstracts and introduction to eliminate unrelated materials.

Criteria for Including or Excluding Studies From the Review

Table 1 provides a framework for describing the eligibility outlining the inclusion and exclusion criteria.

**Table 1:** Inclusion and exclusion criteria

Inclusion criteria	Exclusion criteria
Articles published from 2011 to date (although GammaTile therapy was cleared in 2018, the research into this therapy started many years ago)	Journal articles that do not differentiate GammaTile therapy from traditional radiation therapies
Articles where treatment groups used GammaTile surgically targeted radiation therapy to treat a brain tumor	Articles where treatment groups hardly utilized GammaTile surgically targeted radiation therapy to treat a brain tumor
Peer-reviewed articles containing abstract	Articles that lack controlled groups
Articles that are published in the English language	Articles that lack abstract and those whose full text is unavailable
Articles that compare GammaTile and other traditional radiation therapies in treating brain tumors	

Outline Outcome Measures

One of the fundamental outcome measures of employing GammaTile surgically targeted radiation therapy for brain tumors is an effective and safe, cost-effective therapy for the brain tumor. Another outcome measure for using GammaTile therapy is eliminating the need for repeated treatment after tumor resection. Also, the global improvement in access to care and guaranteeing 100% compliance was another fundamental outcome measure for this study.

Quality Evaluation of Eligible Articles

The study used the Jadad score to evaluate the literature quality by assessing the randomization, blinding, random sequence, and dropouts. In this respect, literature articles with Jadad scores between 1 and 3 are considered low-quality research. On the other hand, literature materials between 4 and 7 are regarded to be high-quality research.

Data Extraction and Analysis

Data will be extracted as intent-to-treat (ITT), randomized using each follow-up data. In this respect, Stata 12.0 (StataCorp LLC, College Station, Texas, USA) and RevMan 5.0 were employed to analyze all the identified articles. Stata 12.0 helped assess the publication bias and sensitivity analysis, while RevMan 5.0 was utilized to compute the collective effect size. The researchers used 95% confidence interval (CI) and relative risk (RR) to analyze dichotomous data and standardized mean differences to analyze continuous variable data.

Similarly, the 12 indexes were employed to analyze heterogeneity. Therefore, I2 was less than 25%, and there was low heterogeneity, thus requiring the fixed-effects model application to group effect size. Also, where the I2 index was more than 25% but less than 50%, there was moderate heterogeneity. Equally, where the I2 index was more than 50%, there was significant heterogeneity. Therefore, the random-effects model was applied to group effect size to achieve more traditional results.

Furthermore, the researcher also performed sensitivity analysis where heterogeneity was significant to identify the heterogeneity source and assess the outcomes' robustness. Also, the researcher employed the funnel plot to qualitatively evaluate publication bias and used Begg's test to assess the publication bias quantitatively. Consequently, where the publication bias was regular and the P-value was more significant than 0.05, there was no publication bias.

Results

**Figure 1:**
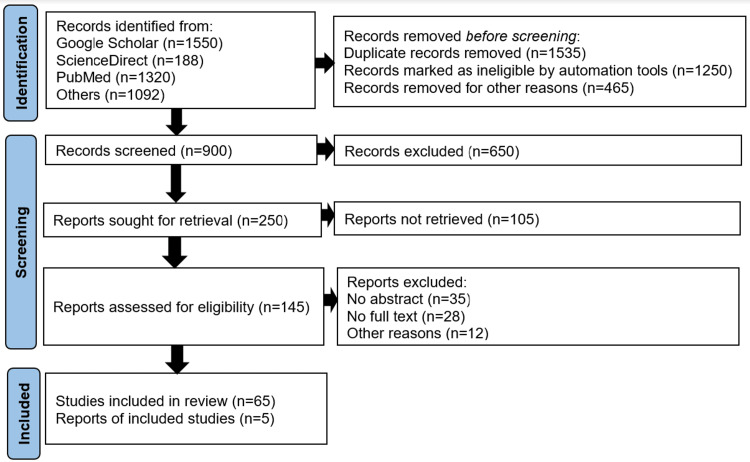
PRISMA flow diagram showing study identification and database PRISMA: Preferred Reporting Items for Systematic Reviews and Meta-Analyses

The researcher identified a total of 4,150 articles based on the selected search strategy. Out of these 4,150 articles, the researcher retrieved 900 articles because they appeared to be related to the study topic. From these 900 retrieved articles, the researcher excluded 650 journal articles for various reasons, such as abstract screening using keywords, thus retrieving 250 citations. The researcher further applied the exclusion/inclusion criteria to these retrieved articles by screening their full text and further excluded 180 articles. Consequently, the researcher retrieved 70 citations in this comprehensive literature review, as shown in the Preferred Reporting Items for Systematic Reviews and Meta-Analyses (PRISMA) diagram in Figure 1.

Properties of GammaTile

GammaTile is a newly US FDA-cleared device incorporating Cs-131 radiation that emits seeds in absorbable collagen-based carrier tile for surgically targeted radiation therapy to attain highly conformal radiation at the time of surgery [17]. Moreover, since GammaTile is a form of brachytherapy, it is vital to differentiate it from other forms of brachytherapy. Glioblastoma is the most prevalent primary malignant neoplasm among the adults' central nervous systems (CNS) [2]. It is linked to significant mortality and morbidity [18]. The foundation therapy is the neurosurgical resection's multimodal approach followed by concurrent chemoradiation therapy administration from four to eight weeks after the first surgery [19]. It is important to note that most glioblastoma reappearance or development occurs locally, directly next to the resection cavity [20].

Autopsy and surgical studies have shown that microscopic tumor cells extend at least 2 cm away from the noticeable tumor [3]. Consequently, studies have shown that delays in commencing radiation therapy beyond recovery are linked to poor survival outcomes. Furthermore, other studies also show that most reappearances happen near the resection cavity for patients who commence concurrent chemotherapy and radiation therapy within 4-8 weeks of recovery time [20]. In this respect, there was a need to develop therapeutic platforms that could supplement the local control cavity to enhance clinical results. Brachytherapy became the most attractive option to help augment local control as it entails the implantation of intracavitary or interstitial radioactive sources nearby the target tissue [21]. The natural radioactivity's initial discovery led to the proposal of brachytherapy, which continues to be a vital therapeutic platform for breast, prostate, ocular, gynecologic, and other non-CNS neoplasms [5]. The radioactivity emitting sources used earlier have been substituted with more effective and safer isotopes that can deliver a more targeted dose [22]. The invention of GammaTile, a device containing Cs radiation-emitting seeds entrenched in the resorbable collagen-based carrier tile, has been instrumental in reducing the technical obstacles to surgical application and radiation planning [23].

GammaTile therapy differs from other types of radiation treatment in various ways. With conventional radiation treatments, neurosurgeons remove the brain tumor, and patients get radiation delivered by the outside ray from the external body [24]. Moreover, in this conventional radiation approach, patients undergo as many as 30 treatments extending over six weeks plus chemotherapy [24]. However, with GammaTile surgically targeted radiation therapy, the neurosurgeon places the tiles containing radiation seeds directly into the brain cavity where the brain tumor grew, which is the location most likely to reappear [25]. Surgeons place small radioactive seeds into the patient to kill cancer cells and shrink tumors. Therefore, the difference between GammaTile therapy and other radiation treatments is that the collagen tiles in GammaTile therapy provide the buffer between the radiation seeds and the surrounding tissue [26]. This approach helps prevent the radiation seeds from damaging healthy brain tissue.

Table 2 contains data demonstrating GammaTile's favorable safety and efficacy profile. Genetically, GammaTile is a surgically targeted radiation therapy (STaRT) for brain tumors, such as brain metastases, high-grade gliomas, and recurrent meningiomas [27]. Table 2 seeks to review the technical specifications of the GammaTile surgically targeted radiation therapy and describe the preliminary clinical experiences and deliberate opportunities and shortcomings relating to the STaRT's clinical translation as glioblastoma therapy.

**Table 2:** Studies evaluating GammaTile surgically targeted radiation therapy for brain tumors OS: overall survival; FFP: freedom from progression; rBrM: recurrent brain metastasis; GT: GammaTile; CSF: cerebrospinal fluid; SRS: stereotactic radiosurgery; rSRS: repeat stereotactic radiosurgery; GBM: glioblastoma; DBF: distant brain failure; PSRT: post-salvage radiation therapy; RN: radiation necrosis; SR: salvage resection; Cs-131: cesium-131; n/a: not available/applicable

Study	Year	Tumor patients	Patients	Local FFP	Distant FFP	Median OS	One-year OS	Complications (total %)
Wernicke et al. [21]	2014	24	Brain metastasis	93.8% (1 year)	48.4% (1 year)	9.9 months	50%	CSF leak, infection, seizure (12.5%)
Pham et al. [22]	2015	24	Brain metastasis	93.8% (1 year)	48.4% (1 year)	9.9 months	50%	CSF leak, infection, seizure (12.5%)
Wernicke et al. [24]	2017	42	Brain metastasis	89% (1 year)	52% (1 year)	15.1 months	58%	Seizure, infection, CSF leak (26%)
Wernicke et al. [24]	2016	13	Brain metastasis	83.3% (1 year)	46.7% (1 year)	7.7 months	24.7%	Infection, pseudomeningocele, seizure, asymptomatic radionecrosis (46%)
Brachman et al. [25]	2018	19	Recurrent meningioma	Not reached	n/a	26 months	Not reported	Alopecia, seizure, radionecrosis, hygroma, infection (36%)
Brachman et al. [25]	2018	74	Previously radiated brain tumor	Reported as local control	Not reported	n/a	50%	Infection, CSF leak, hematoma, shunt placement, coma, radionecrosis (17%)
Gessler et al. [26]	2020	16	MGMT unmethylated (MGMTu)	86% (6 months)	8 months	20 months	55%	One 30-day re-admission (4.5%) for an incisional cerebrospinal fluid leak,
Gessler et al. [26]	2021	6	Methylguanine-DNA methyltransferase methylated (MGMTm)	81% (12 months)	8 months	37.4 months	n/a	One 30-day re-admission (4.5%) for an incisional cerebrospinal fluid leak,
Palmisciano et al. [27]	2022	176	brain metastases	94% (1 year)	53.5% (1 year)	16.2 months	n/a	Post-treatment radiation necrosis, seizure, and surgical wound infection occurred in 3.4% of patients
Palmisciano et al. [27]	2022	65	high-grade gliomas	94% (1 year)	53.5% (1 year)	16.2 months	n/a	Post-treatment radiation necrosis, seizure, and surgical wound infection occurred in 4.7% of patients
Palmisciano et al. [27]	2022	38	meningiomas	94% (1 year)	53.5% (1 year)	16.2 months	24%	Post-treatment radiation necrosis, seizure, and surgical wound infection occurred in 4.3% of patients
Warren et al. [28]	2021	5	gliomas	Reported as local control	Not reported	2.9 months	33%	One patient had a delayed epidural hematoma requiring reoperation, unrelated to GT implantation.
Warren et al. [28]	2021	5	meningiomas	Reported as local control	Not reported	4.8 months	n/a	One patient had a delayed epidural hematoma requiring reoperation, unrelated to GT implantation.
Warre et al. [28]	2021	2	metastatic tumors		Not reported	5.8 months	33.3%	One patient had a delayed epidural hematoma requiring reoperation, unrelated to GT implantation.
Imber et al. [29]	2022	20	post-stereotactic radiosurgery (SRS) rBrM	Reported as local control	4% (1 year)	1.9-11.7 months	n/a	There was one postoperative wound dehiscence
Budnick et al. [3]	2021	7	Recurrent glioblastoma multiforme (GBM)	88% (1 year)	89% (1 year)	18 months,	n/a	Radiation necrosis, residual tumor, second resection to some patients
Nakaji et al. [30]	2020	11	12 recurrent brain tumors and 4 previous untreated	83% (1 year)	n/a	9.3 months	n/a	Grade 2 and grade 3 radiation brain changes in 2 tumor beds
Arsenault et al. [31]	2021	1	Brain metastasis	81.6% (1 year)	51.8% (1 year)	n/a	n/a	Developed seizures and headaches,
Easwaran et al. [32]	2021	1	Glioblastoma	Not reached	Not reported	n/a	n/a	The patient tolerated the procedure without complication and was discharged home on a postoperative day one.
O'Connell et al. [33]	2019	1024	Brain metastases (BM)	Reported as local control	Not reported	n/a	n/a	The patients tolerated the procedure without complication
Moreau et al. [34]	2018	30	Brain metastases	82.9% (1 year and 6 months)	67.8% (1 year and 6 months)	14.2 months	n/a	Concerning toxicities, edema, radionecrosis, and hemorrhages were identified in some patients
Ebner et al. [35]	2017	294	Brain metastases	68% (1 year)	48% (1 year)	12 months	n/a	The patients tolerated the procedure without complication
Holt et al. [36]	2015	13	Brain metastases	75% (1 year)	Not reported	9 months	13.3%	two patients developed DBF after rSRS, 2 resulted in either grade 2 radionecrosis with grade 3 seizures or grade 3 radionecrosis
Wilcox et al. [37]	2021	135	Recurrent brain metastases (rBrM)	40.2% (1 year)	Not reported	13.4 months	95%	SR + PSRT was associated with an increased risk of radiographic RN at 12 months
Raleigh et al. [38]	2017	95	Brain metastases	90% (1 year)	Not reported	62.3 months	n/a	The patients tolerated the procedure without complication

About half of the patients treated for brain tumors tend to experience tumor recurrence within one year, which is a scary statistic. However, with this new type of radiation therapy developed to treat recurrent brain tumors called surgically targeted radiation therapy, there is hope that these statistics will come down [26]. Most of the studies in Table 1 have proved that GammaTile, an FDA-cleared surgically targeted radiation therapy, can prevent or delay tumor cells from multiplying and developing the recurrent tumor [27]. This therapy targets residual tumor cells with radiation before they can extensively reproduce [29].

Traditionally, the standard-of-care treatment for brain tumors involves surgery, and after several weeks, it is followed by many hospital visits for the traditional radiation. However, with GammaTile surgically targeted radiation therapy, brain tumor treatment happens immediately after removing the tumor [29]. This radiation starts to instantly treat the brain after surgery to remove the brain tumor. The radiation immediately starts to treat the location next to where the tumor was removed, thus preventing recurrences [3]. The placement of the radiation capsule takes about five minutes, and the patient is guaranteed to receive 90% of the radiation in 33 days. In 100 days, the patient will have received all the radiation [31].

Advantages of GammaTile Surgically Targeted Radiation Therapy

One of the fundamental advantages of the GammaTile is that it delivers the radiation directly where it is required and averts radiation to the entire body, which helps protect healthy tissue [31]. Also, the GammaTile surgically targeted radiation therapy helps enhance the patient experience by offering additional benefits for the patient's quality of life [32]. The standard treatments for preventing brain tumor recurrence usually include external beam radiation therapy (EBRT). Based on this standard treatment therapy, patients must visit hospitals for their daily treatment for weeks [33]. Furthermore, patients have to wait weeks after the tumor's removal to start EBRT treatment, which is likely to enable residual tumor cells to reappear [34].

Furthermore, in external beam radiation therapy, the radiation beam often travels via healthy tissue, thus heightening the risk of harming non-tumorous tissue [35]. On the other hand, the surgically targeted radiation therapy is localized to restrict radiation delivery to tumor-affected parenchyma [36]. In this respect, the localized delivery helps reduce possible side effects and neurocognitive decline linked to external beam radiation therapy EBRT [36]. Similarly, surgically targeted radiation therapy reduces the likelihood of treatment associated with hair loss, which is the case with EBRT [37,38]. Table 3 outlines studies combining brachytherapy with other standard-of-care treatments.

**Table 3:** Studies combining brachytherapy with other standard-of-care treatments GBM: glioblastoma; HDR-ICBT: high-dose-rate intracavitary brachytherapy; RT: radiotherapy; 5-ALA: 5-aminolevulinic acid; EBRT: external beam radiation therapy; IBT: intraluminal brachytherapy; HDBT: high-dose brachytherapy; PB: prostate seed brachytherapy; OS: overall survival; PFS: progression-free survival; LFTs: liver function tests; HDR: high dose rate; CCRT: concurrent chemoradiotherapy; BF: biochemical failure; IC-IS: intracavitary and interstitial

Study	Year	Patients (number)	Tumor/cancer	Treatment	Median OS	PFS	Complications (%)
Chen et al. [5]	2007	18	Newly diagnosed GBM	Resection, ^125^IBT, and postoperative RT	28.5 months	14.25 months	The study terminated early due to high toxicity, radionecrosis, intracranial hemorrhage, infection, and deep vein thrombosis (61%)
Waters et al. [6]	2013	11	Newly diagnosed GBM	Resection, GliaSite (^125^I) or MammoSite (^192^Ir), postoperative radiation therapy, and temozolomide	15.6 months	10 months	Seizure, reversible hemiparesis (18%)
Archavlis et al. [7]	2014	17	Recurrent GBM	Reresection with 5-ALA, HDR-BT (^192^Ir), temozolomide	Nine months	Seven months	Thrombocytopenia, leukopenia, increased LFTs, infection, radionecrosis (35%)
Joseph et al. [8]	2020	113	Localized cervical cancer	Primary EBRT and intracavitary brachytherapy	28 months	24 months	Generally, nine patients exhibited documented evidence of grade 3 toxicity, two patients developed grade 3 bladder toxicity, and seven patients developed grade 3 rectal toxicity (16%)
Haseltine et al. [9]	2016	61	Non-melanomatous skin cancers	HDR-BT and EBRT	30 months	23 months	Five of six "poor" cosmetic outcomes and the only grade 3 toxic events were found in the standard fractionation EBRT group (22%)
Zelefsky et al. [10]	2011	729	Prostate cancer	High-dose intensity-modulated radiotherapy and brachytherapy	48.5 months	36 months	Late grade 2 urinary toxicities were more often observed for brachytherapy than intensity-modulated radiotherapy (19.9%)
Korenaga et al. [11]	2022	6,047	Cervical cancer	Treated with chemotherapy and concurrent EBRT as well as brachytherapy	15.3 months	13 months	Seizure, urinary toxicities (29.3%)
Toita et al. [12]	2012	71	Locally advanced uterine cervical cancer	CCRT with HDR-ICBT	28 months	24 months	The two-year cumulative late complication rates for grade 1, grade 2, and grade 3 were recorded (24%)
Song et al. [13]	2020	76	Cervical cancer	Combined external beam radiation therapy and HDR-ICB	60 months	45.2 months	Some patients developed locoregional recurrence, and others developed distant recurrence (47.4%)
Galdos-Bejar et al. [14]	2022	419	Localized prostate cancer	EBRT + HDBT in the region	33.81 months	37.36 month	The EBRT + HDBT group had a 40% lower risk of presenting BF (40%)
Ye et al. [15]	2022	32	Esophageal cancer	EBRT + IBT	19 months	15.3 months	Grade 3 or higher acute side effects included two cases of dysphagia and three cases of bone marrow suppression; severe late side effects included three cases of fistula, three cases of radiation pneumonia, and five cases of stenosis requiring treatment (34%)
Qu et al. [16]	2021	34	Advanced cervical cancer	Intracavitary/interstitial applicator + distal parametrial free needle interstitial brachytherapy	44.5 months	32.8 months	No grade 3 or 4 treatment-related toxicities were observed (0%)
Aggarwal et al. [17]	2015	59	Esophageal carcinoma	Combination of external beam radiotherapy and high-dose-rate brachytherapy	12.3 months	10 months	No grade 3 or 4 treatment-related toxicities were observed (0%)
Mohamed et al. [18]	2015	23	Advanced cervical cancer	IC-IS BT combined with EBRT PB	14.5 months	11.2 months	With the EBRT PB scenario, three patients received high-risk clinical target volume D90 of <79 Gy (13.04%)
Alam et al. [19]	2019	72	Locally advanced carcinoma cervix	Interdigitated HDR-ICBT versus sequential HDR-ICBT with EBRT	10 months	Seven months	Treatment interruption due to treatment-related toxicity was slightly higher in the study group than in the control group, but it was statistically insignificant (15.7%)
Bhuiyan et al. [20]	2014	90	Locally advanced carcinoma of the uterine cervix	External beam radiotherapy and intracavitary brachytherapy	14 months	12.5 months	Ten patients had a positive Pap-smear with clinical signs of persis­tence disease (11.11%)

A summary of the progression-free survival (PFS) and overall survival (OS) from Table 3 is outlined in Table 4. 

**Table 4:** Summary of median PFS and median OS from Table 3 PFS: progression-free survival; OS: overall survival

References	PFS (months)	Median OS (months)
Chen et al. [5]	14.25	28.5
Waters et al. [6]	10	15.6
Archavlis et al. [7]	7	9
Joseph et al. [8]	24	28
Haseltine et al. [9]	23	30
Zelefsky et al. [10]	36	48.6
Korenaga et al. [11]	10	8
Toita et al. [12]	24	28
Song et al. [13]	45.2	60
Galdos-Bejar et al. [14]	37.36	33.81
Ye et al. [15]	15.3	19
Qu et al. [16]	44.5	32.8
Aggarwal et al. [17]	10	12.3
Mohamed et al. [18]	11.2	14.5
Alam et al. [19]	7	10
Bhuiyan et al. [20]	14	12.5

**Figure 2:**
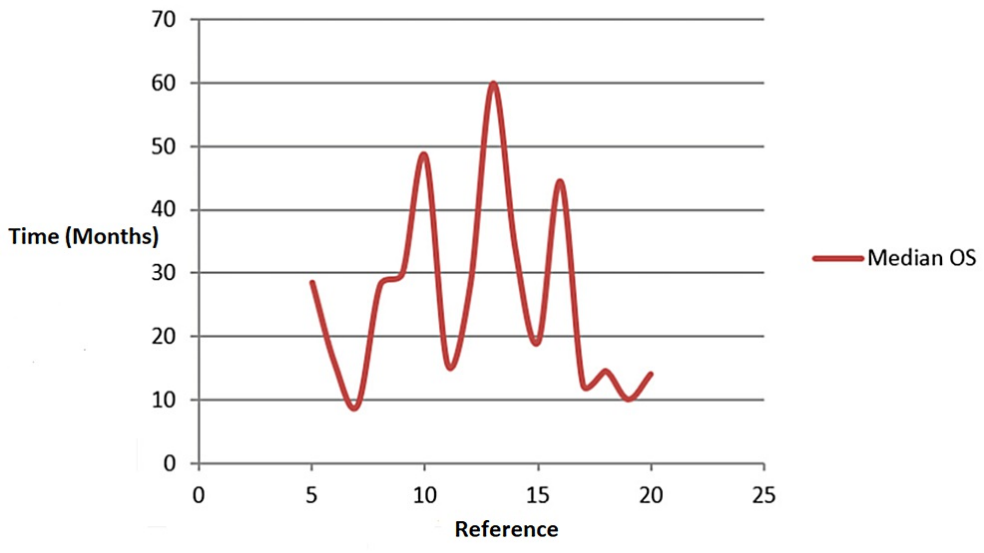
Studies with GT and other treatments versus median OS GT: GammaTile; OS: overall survival

Figure 2 outlines the progression-free survival in months of studies with GammaTile and other treatments. The median overall survival refers to the length of time from the period of diagnosis or the commencement of treatment for the cancer disease. Half of the patients in the group of patients diagnosed with the disease are still alive. In the clinical trial, measuring the median overall survival is an instrumental way to see how well the new treatment works. Figure 1 shows the various median OS periods for GammaTile and other treatment studies. The study by Song et al. (2020) [13] has the highest median OS, asserting that it will take 60 months for half of the patients diagnosed with the cervical cancer group to die. Bhuiyan et al. (2014) [20] established that it takes 14 months for half of the patients diagnosed with locally advanced carcinoma of the uterine cervix to die. It also means that half of the patients diagnosed with locally advanced carcinoma of the uterine cervix will still be alive after 14 months. In the study by Chen et al. (2007) [5], it was found that half of the patients newly diagnosed with GBM will still be active after 28.5 months.

**Figure 3:**
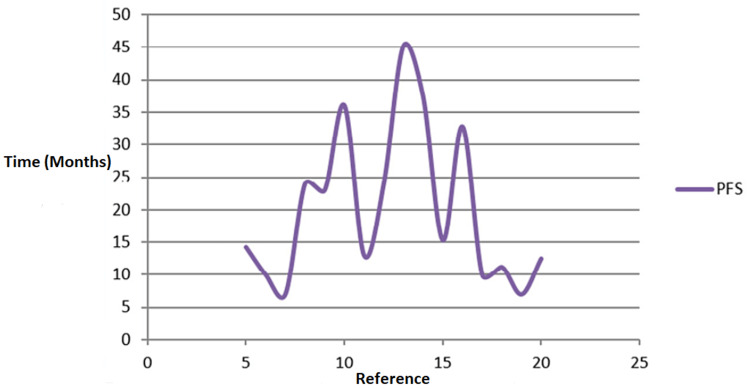
Studies outlining GT and other treatments versus PFS GT: GammaTile; PFS: progression-free survival

Figure 3 outlines studies with GammaTile and other treatments, showing progression-free survival (PFS). Progression-free survival refers to the time during and after treating the disease that patients live with but does not get worse. In the clinical trial, measuring the progression-free survival will be instrumental in helping to see how the GammaTile surgically targeted radiation therapy and other treatment work. Song et al. [13] found that it will take cervical cancer patients 45.2 months during and after the treatment to live with the disease without getting worse. On the other hand, Bhuiyan et al. [20] found that it will take patients diagnosed with locally advanced carcinoma of the uterine cervix 12.5 months during and after the treatment to live with the diseases without getting worse. In the study by Chen et al. [5], they found that it will take 14.25 months for newly diagnosed GBM patients to live with the disease without getting worse.

**Figure 4:**
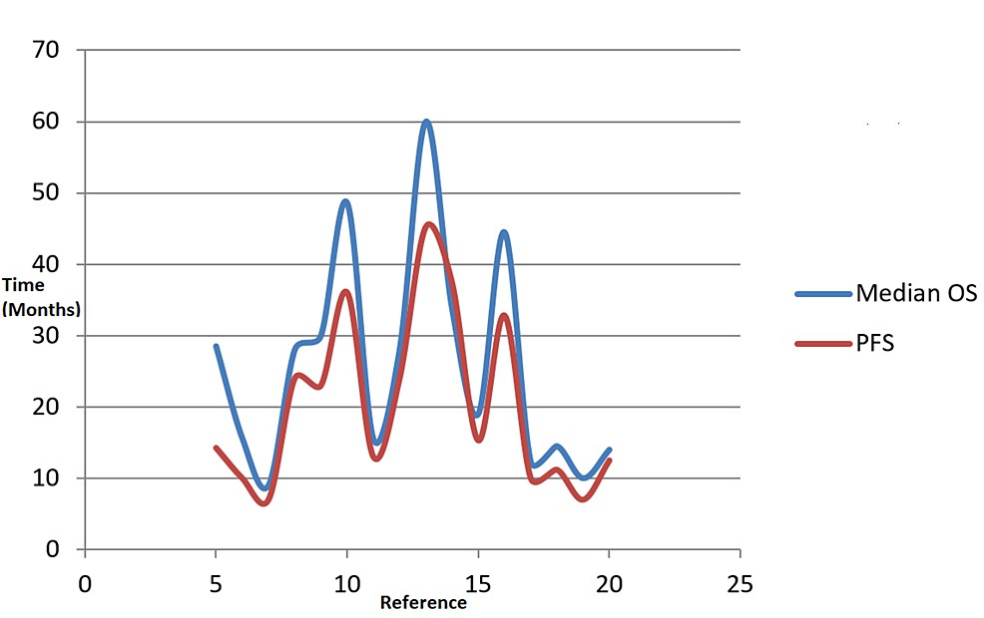
Combination of OS and PFS OS: overall survival; PFS: progression-free survival

Figure 4 shows studies with GammaTile and other treatments versus median OS and PFS. Clinical trials often look at specific outcomes, whereas in some trials, researchers look at the PFS, which measures how long a patient is on the treatment before their cancer starts to grow. Moreover, researchers can also look at the median OS, which measures how long patients live after starting the treatment. Based on these results, the PFS results come in sooner compared to OS, thus making sense for the researcher to use PFS as a surrogate endpoint for the direct measure of how the patient functions, feels, or survives.

GammaTile Implant Procedure and Administration

GammaTile implant procedure and administration involves a constellation of preparation from commissioning, pre-implant, implant, and post-implant. The implant procedure and administration are outlined in Table 5.

**Table 5:** GammaTile implant procedure and administration ADCL: Accredited Dosimetry Calibration Laboratory; OR: operation room; GTV: gross tumor volume; TPS: treatment planning system; Cs-131: cesium-131

Commissioning the program	Pre-implant		GammaTile implant day	Post-implant
Medical physicist	Medical physicist	Radiation oncologist		Medical physicist
Draft radiation safety	1. Prepare written directive and documentation, check GammaTile availability, place an order at least seven days before implant, and confirm trays are sterilized and radiation badges are available and sterilized (two hours); 2. receive GammaTile trays, verify calibration seed, ready badges, sterile trays, and survey meter and patient chart two hours before the implant	Patient consultation for one hour	Craniotomy, tumor resection, intraoperative MRI, GammaTile implant, and surgical closure for 5-9 hours (neurosurgeon)	Log in/out isotope room, and equipment transport to OR (one hour)
ADCL calibration factor for Cs-131 seed (two weeks)		Import preoperative images to TPS and perform segmentation of preoperative GTV volumes for one hour		Post-implant radiation survey and implant documentation for one hour
Commissioning TPS (two weeks)				Release information and signature with patients' family one day after implant in a hospital room for one hour
Approve radiation safety protocols	Estimate number of GammaTiles, measure preoperative GTV volume, estimate area to cover, and discuss with the team for one hour			Postoperative target segmentation for two hours
Radiation safety officer	Medical physicist	Radiation oncologist		Radiation oncologist

GammaTile surgically targeted radiation therapy allows patients to continue with their daily lives while undergoing treatment, as they are not required to keep visiting the hospital for every dose. In this respect, the new radiation therapy treatment plays an instrumental role in guaranteeing 100% compliance [39]. Similarly, with this new type of radiation therapy, patients hardly have to wait weeks to commence radiation treatment after surgery, as the treatment starts immediately after surgery, thus preventing recurrences [36]. Also, the GammaTile is composed of collagen embedded with radiation seeds. The collagen tile helps protect healthy brain tissues from radiation exposure, thus reducing the risk of radiation-related side effects and deterring hair loss [40]. The tile is finally absorbed in the body, thus eliminating the need for another surgical removal.

Table 3 shows the GammaTile implant procedure and administration, providing GammaTile implementation and procedure's projected workflow and time frame. The top of the table, from left to right, shows the timeline of the events, such as program commissioning, pre-implantation, post-implantation, and during the implantation process. Furthermore, it also shows the personnel involved in every listed task. Also, a line connects personnel involved in every task with every task performed. Furthermore, the personnel is listed side by side for the tasks involving over one specialty.

Based on the information in Table 3, medical physicists review radiation safety with patients before the surgery. Furthermore, they determine the tile numbers depending on the anticipated tumor bed's postoperative surface contours and then custom-ordered to ensure they are available in a week. The radiation oncology department receives and handles the tiles based on institutional policy. During surgery, the medical physicist brings the tiles to the operating room and undertakes the radiation safety checks during the implantation process. The medical physicist utilizes the intraoperative MRI to help verify the tumor's maximum safe resection before surgically targeted radiation therapy. Immediately after conducting maximum resection, the radiation oncologist cleans sterilely, unloads, prepares the surgically targeted radiation therapy implant, and hands it to the surgeon [41]. The surgeon places the tiles into the resection cavity under loupe or microscope magnification, and the implantation often takes 2-5 minutes.

Discussion

For many years, clinicians have sought better treatment options for patients suffering from brain tumors that can effectively target tumor cells while protecting the surrounding tissue [42]. Spurred by the available traditional treatment options and the need to improve the standard of care, brain tumor specialists joined together to address this vital need [43]. This group of brain tumor specialists developed GammaTile therapy with medical device specialists. GammaTile therapy refers to the surgically targeted radiation therapy (STaRT) that provides instant, dose-intense treatment after resection [44]. Resection plus GammaTile therapy is instrumental in extending local recurrence-free survival with fewer complications, reduced patient burden, and guaranteed compliance [45]. Therefore, GammaTile therapy guarantees clinical efficiency, immediacy, and efficiency by enabling instant treatment at resection with reduced hospital stay [44]. Moreover, the procedure takes about five minutes to complete tile placement and simplifies care with 100% "built-in" compliance and no special inpatient contraindications or precautions with systemic therapies [46].

GammaTile therapy targets tumor cells while protecting brain tissue. Similarly, the surgically guided treatment of the local radiation dose to the operative bed optimizes the therapeutic margin while reducing complications [47]. Also, the radiation source's structural offset from the brain tissue deters damaging direct seed-to-tissue contact and enables intraoperative modification [48]. Implanting encapsulated Cs radiation emitter seeds in collage-based tiles is vital in lowering the technical barriers linked to conventional brachytherapy [49].

Findings and the Importance of Metrics Gathered From the Results

Compared to other forms of brachytherapy, GammaTile has been found to be one of the most effective treatment options for brain tumors [50]. Brachytherapy's efficacy is often restricted by the flexibility of glioblastoma cell conditions in radiation response and the resistance mechanisms' diversity [51]. However, this treatment is instrumental in offering dose escalation in line with the resection cavity and devoid of any delays associated with surgical recovery. A study by Gessler et al. (2022) [26] compared GammaTile's safety profile after maximum safe resection with published surgical series on glioblastoma resection. The study established that notwithstanding the cohort of patients that went through second and third resection via the same surgical incision, they hardly observed any wound infection in the cohort [26,52].

Furthermore, some studies have sought to compare the duration of hospital stay and operative mortality/mobility for the GammaTile-treated cohort with those who reported glioblastoma patients who went through resection without GammaTile implant [52-54]. Interestingly, no patient in this cohort reported suffering from adverse radiation effects (AREs) to require surgical or medical intervention [52,54]. More importantly, the studies provide outcome and safety data that is more favorable and supports the adoption of GammaTile as an ideal component of the multimodal intervention approach for recurrent glioblastomas [31,55,56].

Moreover, over the past few decades, surgically implanted brachytherapy has demonstrated promise for multiple tumor types [56]. A study by Budnick et al. (2021) [3] established the existence of statistically significant clinical improvement in time to local disease progression relative to a similar patient after their first operation. The study found that at 18 months, the majority of patients had no disease progression; in patients who depicted disease progression, it was established to be 11 months [3]. Generally, studies have established that GammaTile brachytherapy provides sufficient radiation dose treatment to the resection cavities, as depicted in all PTV V100 that were above 80% [57]. Out of the seven patients in the study's cohort, it was established that six patients had their residual tumors well covered [53].

Similarly, the study also found that the disease had developed extensively beyond the preoperatively planned resection location for the patient whose residual tumor had not been well covered. Also, the study reported no complications linked to the GammaTiles' placement and hardly portrayed radiation necrosis on early follow-up imaging [35,58]. Patients receiving GammaTile placement had expected postoperative courses similar to the experience of tumor resection patients, such as hospital and ICU length of stay (LOS), postoperative complications, and release disposition [53]. Consequently, studies have found GammaTile to be a safe type of brachytherapy in recurrent gliomas [59].

A study by Ferreira et al. (2021) [44] sought to help clinicians execute GammaTile brain implant technology. GammaTile can be implanted immediately after resection, which enables it to commence delivering radiation therapy instantly [60]. Based on this study, increasing the time gap between radiation therapy and resection can significantly reduce recurrence-free survival [61]. Consequently, starting radiation therapy immediately after tumor resection can enhance the patients' recurrence-free survival [61]. Moreover, because of the 3 mm seeds' distance from the brain tissue and radionuclide, as well as the Cs' low delivery rate, GammaTile therapy presents a lower risk of radiation injury [44].

Resected brain metastases tend to have a high local recurrence rate without adjuvant therapy [60]. Adjuvant whole-brain radiotherapy (WBRT) is the standard of care, with a local control rate of over 90% [61]. However, adjuvant whole-brain radiotherapy is provided over 10-15 days, thus delaying other therapy, and is linked to acute and long-term toxicities [61]. Consequently, permanent cesium-131 implants can be utilized during metastatic resection to avert the need for extra therapy [21]. Therefore, the study by Wernicke et al. (2014) [21] sought to evaluate the feasibility, efficacy, and safety of the new therapeutic approach with permanent Cs-131 implants during the resection of brain metastases. The study established that the utilization of post-resection permanent Cs-131 brachytherapy implants leads to no radiation necrosis and no local recurrences [61]. Moreover, the study established that post-resection permanent Cs-131 brachytherapy implant is convenient, safe, and well tolerated by patients, leading to a reduced radiation treatment course, minimal toxicity, and a high response rate [62].

Pham et al. (2016) [22] asserted that intraoperative permanent Cs-131 brachytherapy provides a viable alternative to adjuvant whole-brain radiotherapy with negligible toxicity and exceptional response rates. The study by Pham et al. [22] sought to explore the impact of intraoperative Cs-131 on the quality of life and neurocognitive function of patients with resected brain metastases [22]. The study examined newly diagnosed metastasis to the brain treated with Cs-131 brachytherapy seeds after resection to assess their mini-mental status and functional assessment before treatment and again after every 2-6 months with extra follow-up at 12 months [63]. The study established that the patients' mini-mental status examination scores improved significantly after two and 12 months compared to the pre-treatment period. Furthermore, the study also established that brain metastasis patients who received intraoperative permanent Cs-131 brachytherapy implants experienced an improvement in their self-assessment of quality of life and neurocognitive status [64]. Furthermore, besides having excellent local control of metastasis, the study also found that an intraoperative permanent Cs-131 brachytherapy implant is likely to improve brain tumor patients' quality of life and cognitive function [65].

Also, based on the metrics gathered from the results, intraoperative Cs-131 brachytherapy is an effective and promising therapy for large brain metastases that require neurosurgical intervention to offer lower rates of radionecrosis (RN) and enhanced local control as compared to stereotactic radiosurgery to the resection cavity [66]. Similarly, effective treatments for recurrent, previously intracranial meningiomas are restricted, and resection alone can hardly be curative [42]. Combining the maximum safe resection and adjuvant radiation is necessary by utilizing permanent intracranial brachytherapy in patients with recurrent, previously irradiated forceful meningiomas [67]. Also, fundamental external beam radiotherapy coupled with intracavitary brachytherapy is the standard of care for localized cervix carcinoma that is not stable under radical surgery [8].

Furthermore, a study by Bhuiyan et al. [20] sought to evaluate the effectiveness and acute toxicity of four fractions of high-dose-rate intracavity brachytherapy after pelvic external beam radiotherapy in the treatment of locally advanced cervical carcinoma. The study found that high-dose-rate (HDR) brachytherapy in combination with external beam radiotherapy (EBRT) is the safest and most effective treatment for locally advanced carcinoma of the uterine cervix [20]. Also, findings in Table 2 show that interdigitated high-dose-rate intracavitary brachytherapy (HDR-ICBT) tends to have equivalent response and toxicities as chronological high-dose-rate intracavitary brachytherapy (HDR-ICBT) with the benefit of a significant reduction in the overall treatment time (OTT) [19]. The metrics from the results also established that treatment in an entirely radiation-based approach is deliverable with exceptional acquiescence and median survival [17].

Similarly, a study by Qu et al. (2021) [16] explored the dosimetric benefit of combining interstitial/intracavitary applicator with distant parametrial free needle interstitial brachytherapy according to the MRI for locally advanced cervical cancer. The study found that combining interstitial/intracavitary applicator with distant parametrial free needle interstitial brachytherapy provides an effective treatment for cervical cancer patients with distal parametrial extension [16]. Also, the results in Table 2 show that combining intracavitary and interstitial (IC-IS) BT provides a better outcome than using a single treatment approach, as the combination of the approaches was more conformal with minimal regular tissue exposure to intermediate doses [18]. Similarly, Ye et al. (2022) [15] also found that combining external beam radiotherapy (EBRT) with intraluminal brachytherapy (IBT) offers an effective treatment modality for cancer with maximum local control than EBRT alone. These findings were consistent with the findings established in the study by Galdos-Bejar et al. (2022) [14] by asserting that patients treated with the combination of EBRT + HDBT and RP had less biochemical failure and post-treatment toxicity than patients treated with EBRT + HDBT [14].

Also, concurrent chemoradiotherapy (CCRT) utilizing high-dose-rate intracavitary brachytherapy (HDR-ICBT) with low cumulative definitive radiotherapy (RT) attained similar results as those attained with global dose schedules with a lower rate of late toxicity for locally advanced cervical cancer in the Japanese population [12]. Moreover, Korenaga et al. (2022) [11] also sought to evaluate the use of brachytherapy and treatment duration on overall survival for locally advanced cervical cancer. The study found that completing standard-of-care concurrent chemoradiation therapy and brachytherapy within the recommended eight weeks is linked to better overall survival. The study also found that patients who received a brachytherapy boost provided better survival than those who received EBRT alone [11]. Also, Chargari et al. (2017) [57] found brachytherapy to be more effective as part of the conservative strategy for bladder-prostate rhabdomyosarcoma (BP RMS) with comparably low delayed toxicity when compared to previous studies that used external beam radiation therapy. Peach et al. (2021) [60] also found the feasibility of amalgamating GammaTile with dose-matched EBRT volumes in a reproducible way to subtotally resected, recurrent intracranial neoplasms.

The findings also show that brachytherapy that uses permanently implantable collagen tiles that contain Cs-131 helps effectively treat malignant intracranial neoplasms as they have proven seed migration [68]. Furthermore, cesium-131 brachytherapy helped improve local control and provided satisfactory rates of symptomatic adverse radiation effects (AREs) and surgical complications [69]. Imber et al. (2022) [29] found that intraoperative brachytherapy with commercially available Cs-131 implants was linked to better local control and toxicity profiles. Moreover, the results also showed that adjuvant stereotactic radiosurgery significantly helps improve local control with whole-brain radiotherapy as the salvage or adjuvant treatment [70].

Limitations

The researcher noted various limitations, especially when interpreting the results from the different studies. Most of these limitations originate from the nature of the available studies for synthesis. For example, there were significant between-trial variability trial features, such as measures utilized, inclusion criteria, and controlled conditions. Future studies should strive to maintain these variables as consistent as possible in every identified study to ensure more estimates for effect size.

## Conclusions

With the conventional external beam radiation therapy (EBRT) as the adjuvant therapy for brain tumor treatment, access to care is restricted by the substantial fiscal capital investment and the select know-how of radiation oncologists familiar with brain tumor treatment. Moreover, external beam radiation therapy treatments characteristically require patients to travel to the hospital frequently or daily for several weeks. More importantly, patients suffering from recurrent brain tumors who have already received their maximum safe dose of external beam radiation therapy are not likely to have any other treatment options for adjuvant therapy. Also, patients have to wait for several weeks before starting EBRT treatment, thus allowing the possibility of recurrences. In this respect, together with medical device specialists, a group of brain tumor specialists developed GammaTile therapy as an effective and safe radiation option for treating brain tumors.

Neurosurgeon implants GammaTile therapy during surgery at the neurosurgical operating room. GammaTile treatment starts instantly and continues as patients continue with their daily lives. In this respect, GammaTile therapy enhances access to care, guarantees 100% compliance, and eliminates the need for patients to travel to hospitals for radiation treatments regularly. Therefore, based on the metrics found in our results, the GammaTile program's implementation requires collaboration from various specialties, such as radiation oncology, medical physics, and neurosurgery. Furthermore, GammaTile's safe intraoperative implantation calls for wide-ranging preplanning and interdisciplinary partnership.

## References

[1] Penoncello GP, Gagneur JD, Vora SA (2022). Comprehensive commissioning and clinical implementation of GammaTiles STaRT for intracranial brain cancer. Adv Radiat Oncol.

[2] Weinberg J, McAleer MF, Tawbi H, Lang F (2020). A randomized, multicenter Phase III trial of surgery plus stereotactic radiosurgery (SRS) compared with surgery plus permanently implanted collagen tile brachytherapy (CTBT) for resectable metastatic brain tumors-protocol in progress. Neurooncol Adv.

[3] Budnick HC, Richardson AM, Shiue K, Watson G, Ng SK, Le Y, Shah MV (2021). GammaTile for gliomas: a single-center case series. Cureus.

[4] Byrne JD, Botticello T, Niemierko A (2020). Post-operative radiation therapy to the surgical cavity with standard fractionation in patients with brain metastases. Scientific Reports.

[5] Chen AM, Chang S, Pouliot J (2007). Phase I trial of gross total resection, permanent iodine-125 brachytherapy, and hyperfractionated radiotherapy for newly diagnosed glioblastoma multiforme. Int J Radiat Oncol Biol Phys.

[6] Waters JD, Rose B, Gonda DD (2013). Immediate post-operative brachytherapy prior to irradiation and temozolomide for newly diagnosed glioblastoma. J Neurooncol.

[7] Archavlis E, Tselis N, Birn G, Ulrich P, Zamboglou N (2014). Salvage therapy for recurrent glioblastoma multiforme: a multimodal approach combining fluorescence-guided resurgery, interstitial irradiation, and chemotherapy. Neurol Res.

[8] Joseph N, Jayalath H, Balawardena J (2020). Radical external-beam radiotherapy in combination with intracavitary brachytherapy for localized carcinoma of the cervix in Sri Lanka: is treatment delayed treatment denied?. JCO Glob Oncol.

[9] Haseltine JM, Parker M, Wernicke AG, Nori D, Wu X, Parashar B (2016). Clinical comparison of brachytherapy versus hypofractionated external beam radiation versus standard fractionation external beam radiation for non-melanomatous skin cancers. J Contemp Brachytherapy.

[10] Zelefsky MJ, Yamada Y, Pei X, Hunt M, Cohen G, Zhang Z, Zaider M (2011). Comparison of tumor control and toxicity outcomes of high-dose intensity-modulated radiotherapy and brachytherapy for patients with favorable risk prostate cancer. Urology.

[11] Korenaga TK, Yoshida EJ, Pierson W (2022). Better late than never: brachytherapy is more important than timing in treatment of locally advanced cervical cancer. Gynecol Oncol.

[12] Toita T, Kitagawa R, Hamano T (2012). Phase II study of concurrent chemoradiotherapy with high-dose-rate intracavitary brachytherapy in patients with locally advanced uterine cervical cancer: efficacy and toxicity of a low cumulative radiation dose schedule. Gynecol Oncol.

[13] Song J, Alyamani N, Bhattacharya G, Le T, E C, Samant R (2020). The impact of high-dose-rate brachytherapy: measuring clinical outcomes in the primary treatment of cervical cancer. Adv Radiat Oncol.

[14] Galdos-Bejar M, Belanovic-Ramirez I, Alvarado GF, Del Castillo R (2022). Biochemical failure and toxicity in treatment with brachytherapy and external beam radiotherapy compared with radical prostatectomy in localized prostate cancer. Rep Pract Oncol Radiother.

[15] Ye M, Han D, Mao Z, Cheng G (2022). A prospective study of radical external beam radiotherapy versus external beam radiotherapy combined with intraluminal brachytherapy for primary esophageal cancer. Brachytherapy.

[16] Qu HD, Han DM, Zhang N, Mao Z, Cheng GH (2020). Intracavitary/interstitial applicator plus distal parametrial free needle interstitial brachytherapy in locally advanced cervical cancer: a dosimetric study. Front Oncol.

[17] Aggarwal A, Harrison M, Glynne-Jones R, Sinha-ray R, Cooper D, Hoskin PJ (2015). Combination external beam radiotherapy and intraluminal brachytherapy for non-radical treatment of oesophageal carcinoma in patients not suitable for surgery or chemoradiation. Clin Oncol (R Coll Radiol).

[18] Mohamed S, Kallehauge J, Fokdal L, Lindegaard JC, Tanderup K (2015). Parametrial boosting in locally advanced cervical cancer: combined intracavitary/interstitial brachytherapy vs. intracavitary brachytherapy plus external beam radiotherapy. Brachytherapy.

[19] Alam N, Akram M, Siddiqui SA, Hussain MA (2019). Interdigitated versus sequential high-dose-rate intracavitary brachytherapy with external beam radiotherapy in locally advanced carcinoma cervix. J Cancer Res Ther.

[20] Bhuiyan M, Rahman A, Alam S, Shaheen S, Islam M, Mostafa S (2014). External beam radiotherapy and intracavitary brachytherapy is an acceptable treatment for locally advanced carcinoma of the uterine cervix. Bangabandhu Sheikh Mujib Med Univ J.

[21] Wernicke AG, Yondorf MZ, Peng L (2014). Phase I/II study of resection and intraoperative cesium-131 radioisotope brachytherapy in patients with newly diagnosed brain metastases. J Neurosurg.

[22] Pham A, Yondorf MZ, Parashar B (2016). Neurocognitive function and quality of life in patients with newly diagnosed brain metastasis after treatment with intra-operative cesium-131 brachytherapy: a prospective trial. J Neurooncol.

[23] Wernicke AG, Hirschfeld CB, Smith AW (2017). Clinical outcomes of large brain metastases treated with neurosurgical resection and intraoperative cesium-131 brachytherapy: results of a prospective trial. Int J Radiat Oncol Biol Phys.

[24] Wernicke AG, Lazow SP, Taube S (2016). Surgical technique and clinically relevant resection cavity dynamics following implantation of cesium-131 (CS-131) brachytherapy in patients with brain metastases. Oper Neurosurg (Hagerstown).

[25] Brachman DG, Youssef E, Dardis CJ (2018). Resection and permanent intracranial brachytherapy using modular, biocompatible cesium-131 implants: results in 20 recurrent, previously irradiated meningiomas. J Neurosurg.

[26] Gessler DJ, Neil EC, Shah R (2022). GammaTile® brachytherapy in the treatment of recurrent glioblastomas. Neurooncol Adv.

[27] Palmisciano P, Haider AS, Balasubramanian K, D'Amico RS, Wernicke AG (2022). The role of cesium-131 brachytherapy in brain tumors: a scoping review of the literature and ongoing clinical trials. J Neurooncol.

[28] Warren KT, Boucher A, Bray DP (2021). Surgical outcomes of novel collagen tile cesium brachytherapy for recurrent intracranial tumors at a tertiary referral center. Cureus.

[29] Imber BS, Young RJ, Beal K (2022). Salvage resection plus cesium-131 brachytherapy durably controls post-SRS recurrent brain metastases. J Neurooncol.

[30] Nakaji P, Smith K, Youssef E (2020). Resection and surgically targeted radiation therapy for the treatment of larger recurrent or newly diagnosed brain metastasis: results from a prospective trial. Cureus.

[31] Arsenault T, Labak CM, Chaung K (2021). Treating recurrent brain metastases using GammaTile brachytherapy: a case report and dosimetric modeling method. Cureus.

[32] Easwaran TP, Sterling D, Ferreira C (2021). Rapid interval recurrence of glioblastoma following gross total resection: a possible indication for GammaTileⓡ brachytherapy. Cureus.

[33] O'Connell K, Romo CG, Grossman SA (2019). Brain metastases as a first site of recurrence in patients on chemotherapy with controlled systemic cancers: an increasingly urgent clinical scenario. J Clin Oncol.

[34] Moreau J, Khalil T, Dupic G (2018). Second course of stereotactic radiosurgery for locally recurrent brain metastases: safety and efficacy. PLoS One.

[35] Ebner DK, Gorovets D, Rava P, Cielo D, Kinsella TJ, DiPetrillo TA, Hepel JT (2017). Patients with long-term control of systemic disease are a favorable prognostic group for treatment of brain metastases with stereotactic radiosurgery alone. World Neurosurg.

[36] Holt DE, Gill BS, Clump DA (2015). Tumor bed radiosurgery following resection and prior stereotactic radiosurgery for locally persistent brain metastasis. Front Oncol.

[37] Wilcox JA, Brown S, Reiner AS (2021). Salvage resection of recurrent previously irradiated brain metastases: tumor control and radiation necrosis dependency on adjuvant re-irradiation. J Neurooncol.

[38] Raleigh DR, Seymour ZA, Tomlin B (2017). Resection and brain brachytherapy with permanent iodine-125 sources for brain metastasis. J Neurosurg.

[39] McClelland S, Gardner UG, Le Y, Ng SK, Shah MV, Watson GA (2021). Safety and efficacy of GammaTile intracranial brachytherapy implanted during awake craniotomy. Brachytherapy.

[40] Williams VM, Kahn JM, Thaker NG (2021). The case for brachytherapy: why it deserves a renaissance. Adv Radiat Oncol.

[41] Botros D, Dux H, Price C, Khalafallah AM, Mukherjee D (2021). Assessing the efficacy of repeat resections in recurrent glioblastoma: a systematic review. Neurosurg Rev.

[42] Goldman DA, Hovinga K, Reiner AS, Esquenazi Y, Tabar V, Panageas KS (2018). The relationship between repeat resection and overall survival in patients with glioblastoma: a time-dependent analysis. J Neurosurg.

[43] van Linde ME, Brahm CG, de Witt Hamer PC (2017). Treatment outcome of patients with recurrent glioblastoma multiforme: a retrospective multicenter analysis. J Neurooncol.

[44] Ferreira C, Sterling D, Reynolds M, Dusenbery K, Chen C, Alaei P (2021). First clinical implementation of GammaTile permanent brain implants after FDA clearance. Brachytherapy.

[45] Greenwald J, Taube S, Yondorf MZ, Smith A, Sabbas A, Wernicke AG (2019). Placement of 131Cs permanent brachytherapy seeds in a large combined cavity of two resected brain metastases in one setting: case report and technical note. J Contemp Brachytherapy.

[46] Sharanek A, Burban A, Laaper M, Heckel E, Joyal JS, Soleimani VD, Jahani-Asl A (2020). OSMR controls glioma stem cell respiration and confers resistance of glioblastoma to ionizing radiation. Nat Commun.

[47] Brachman D, Youssef E, Dardis C, Smith K, Pinnaduwage D, Nakaji P (2019). Surgically targeted radiation therapy: safety profile of collagen tile brachytherapy in 79 recurrent, previously irradiated intracranial neoplasms on a prospective clinical trial. Brachytherapy.

[48] Rogers L, Nakaji P, Youssef E (2020). Resection and surgically targeted radiation therapy for initial or salvage treatment of aggressive meningioma: results from a prospective trial. Neurosurgery.

[49] Brachman D, Nakaji P, Smith K, Youssef E, Thomas T, Pinnaduwage D, Rogers CL (2020). A prospective trial of resection plus surgically targeted radiation therapy for brain metastasis. Neurooncol Adv.

[50] Han DY, Ma L, Braunstein S, Raleigh D, Sneed PK, McDermott M (2018). Resection cavity contraction effects in the use of radioactive sources (1-25 versus CS-131) for intra-operative brain implants. Cureus.

[51] Gaspar LE, Fisher BJ, Macdonald DR (1992). Supratentorial malignant glioma: patterns of recurrence and implications for external beam local treatment. Brachytherapy.

[52] Gessler DJ, Ferreira C, Dusenbery K, Chen CC (2020). GammaTile®: surgically targeted radiation therapy for glioblastomas. Future Oncol.

[53] Alshammari M, Aljohani MA, Hashash JM (2021). Shwachman-Diamond syndrome in a child presenting with chronic diarrhea: a rare case in family medicine practice. Cureus.

[54] Morgan TM, Press RH, Cutrell PK (2018). Brachytherapy for localized prostate cancer in the modern era: a comparison of patient-reported quality of life outcomes among different techniques. J Contemp Brachytherapy.

[55] Pinzi V, Landoni V, Cattani F, Lazzari R, Jereczek-Fossa BA, Orecchia R (2019). IMRT and brachytherapy comparison in gynaecological cancer treatment: thinking over dosimetry and radiobiology. Ecancermedicalscience.

[56] Lee DJ, Barocas DA, Zhao Z (2018). Contemporary prostate cancer radiation therapy in the United States: patterns of care and compliance with quality measures. Pract Radiat Oncol.

[57] Chargari C, Haie-Meder C, Guérin F (2017). Brachytherapy combined with surgery for conservative treatment of children with bladder neck and/or prostate rhabdomyosarcoma. Int J Radiat Oncol Biol Phys.

[58] Munier S, Ginalis EE, Patel NV (2020). Radiation Necrosis in Intracranial Lesions.

[59] Barbour AB, Jacobs CD, Williamson H, Floyd SR, Suneja G, Torok JA, Kirkpatrick JP (2020). Radiation therapy practice patterns for brain metastases in the United States in the stereotactic radiosurgery era. Adv Radiat Oncol.

[60] Peach MS, Burke AM, Jo J, Ju AW, Yang K (2021). Gammatile brachytherapy combined with external beam radiation therapy for the treatment of a partially resected secondary glioblastoma (WHO grade 4 IDH-mutant astrocytoma): matching external beam dose gradient to brachytherapy dose fall-off. Cureus.

[61] Mooney MA, Bi WL, Cantalino JM (2020). Brachytherapy with surgical resection as salvage treatment for recurrent high-grade meningiomas: a matched cohort study. J Neurooncol.

[62] Rogers L, Zhang P, Vogelbaum MA (2018). Intermediate-risk meningioma: initial outcomes from NRG Oncology RTOG 0539. J Neurosurg.

[63] Pinnaduwage DS, Srivastava SP, Yan X, Jani S, Brachman DG, Sorensen SP (2022). Dosimetric impacts of source migration, radioisotope type, and decay with permanent implantable collagen tile brachytherapy for brain tumors. Technol Cancer Res Treat.

[64] Chen WC, Lafreniere M, Phuong C (2022). Resection with intraoperative cesium-131 brachytherapy as salvage therapy for recurrent brain tumors. J Neurosurg.

[65] Amsbaugh MJ, Boling W, Woo S (2015). Tumor bed radiosurgery: an emerging treatment for brain metastases. J Neurooncol.

[66] Ciezki JP, Weller M, Reddy CA (2017). A comparison between low-dose-rate brachytherapy with or without androgen deprivation, external beam radiation therapy with or without androgen deprivation, and radical prostatectomy with or without adjuvant or salvage radiation therapy for high-risk. Int J Radiat Oncol Biol Phys.

[67] Tanderup K, Ménard C, Polgar C, Lindegaard JC, Kirisits C, Pötter R (2017). Advancements in brachytherapy. Adv Drug Deliv Rev.

[68] Ostrom QT, Gittleman H, Xu J, Kromer C, Wolinsky Y, Kruchko C, Barnholtz-Sloan JS (2016). CBTRUS statistical report: primary brain and other central nervous system tumors diagnosed in the United States in 2009-2013. Neuro Oncol.

[69] Mahajan A, Ahmed S, McAleer MF (2017). Post-operative stereotactic radiosurgery versus observation for completely resected brain metastases: a single-centre, randomised, controlled, phase 3 trial. Lancet Oncol.

[70] Mahase SS, Navrazhina K, Schwartz TH, Parashar B, Wernicke AG (2019). Intraoperative brachytherapy for resected brain metastases. Brachytherapy.

